# Synthetic Hydroxyapatite Inhibits Bisphosphonate Toxicity to the Oral Mucosa In Vitro

**DOI:** 10.3390/ma13092086

**Published:** 2020-05-01

**Authors:** George Bullock, Cheryl Miller, Alasdair McKechnie, Vanessa Hearnden

**Affiliations:** 1 Department of Materials Science and Engineering, The University of Sheffield, Sheffield S3 7HQ, UK; g.d.bullock@sheffield.ac.uk (G.B.); v.hearnden@sheffield.ac.uk (V.H.); 2 School of Clinical Dentistry, The University of Sheffield, Sheffield S10 2TA, UK; 3 School of Dentistry, University of Leeds, Leeds LS2 9JT, UK; a.mckechnie@leeds.ac.uk

**Keywords:** osteonecrosis, osteonecrosis of the jaw, fibroblasts, keratinocytes, pamidronic acid, pamidronate, zoledronic acid, zoledronate, BRONJ, MRONJ

## Abstract

Medication-related osteonecrosis of the jaw (MRONJ) is a side effect of bisphosphonate therapy, characterised by exposed necrotic bone. The soft tissues of the oral mucosa no longer provide a protective barrier and MRONJ patients experience pain, infections and difficulties eating. We hypothesised that hydroxyapatite (Ca_5_(PO_4_)_3_(OH)) could reduce bisphosphonate concentrations and protect the oral mucosa by exploiting bisphosphonate’s calcium binding affinity. The effect of zoledronic acid (ZA) and pamidronic acid (PA) on the metabolism of oral fibroblasts, oral keratinocytes and three-dimensional oral mucosa models was investigated and then repeated in the presence of hydroxyapatite granules. Without hydroxyapatite, ZA and PA significantly reduced the metabolic activity of oral cells in a dose-dependent manner. Both drugs reduced epithelial thickness and 30 µM ZA resulted in loss of the epithelium. Hydroxyapatite granules had a protective effect on oral cells, with metabolic activity retained. Oral mucosa models retained their multi-layered epithelium when treated with ZA in the presence of hydroxyapatite granules and metabolic activity was comparable to controls. These results demonstrate hydroxyapatite granules protected oral soft tissues from damage caused by bisphosphonate exposure. Porous hydroxyapatite granules are currently used for socket preservation and this data suggests their potential to prevent MRONJ in at-risk patients.

## 1. Introduction

Medication-related osteonecrosis of the jaw (MRONJ) is a disease defined by jawbone necrosis and exposure through the overlying soft tissue. The exposed bone causes pain, is prone to recurrent infection and leads to difficulty with eating, drinking and speech [1]. MRONJ commonly develops in patients following wounding of the jaw and, in the majority of cases, follows a tooth extraction. The medications most commonly associated with MRONJ are bisphosphonates (BPs). However, other anti-resorptives and anti-angiogenic medications have been linked to the disease [2]. Incidence is variable depending on the medication used, therapy length and other risk factors including poor oral hygiene. The risk of developing MRONJ ranges from 1% to 10% for patients prescribed BPs for bone metastases, and 0.01% to 0.001% for those treated for osteoporosis [3]. MRONJ is currently without an effective treatment [2].

BPs act either by inhibition of adenosine triphosphate-dependent metabolic pathways or the mevalonate pathway, depending on their chemical structure, ultimately leading to cell death [4,5]. BPs bind to the calcium in hydroxyapatite avidly, causing a preferential effect on the osteoclasts in bone, and have a prolonged effect once administered [5,6]. Some BPs have half-lives in bone measured in years, so ceasing treatment after MRONJ develops or withholding BP treatment prior to dental work have shown little benefit.

Since it was first described in 2003, devising a treatment for MRONJ has been challenging due to the relatively low incidence and incomplete knowledge of its pathogenesis [7]. However, incidence rates are rising [8]. Bone exposure through the soft tissue is the primary clinical feature of MRONJ and closure of the MRONJ wound has been hypothesised as key to treatment [9]. While most studies agree that clinically relevant BP concentrations are toxic to oral mucosa cells [10,11,12,13], there is a lack of consensus on the concentration at which this toxicity occurs, and one study has shown that low levels of BPs can have a positive effect on keratinocytes [14]. Most work has focused on cells in monolayer, with few studies investigating how BPs affect the oral mucosa in 3D [15,16,17,18]. It is therefore important to further examine this toxicity in a more comprehensive manner.

Several strategies have been investigated to reduce BP action at the wound site. Elsayed et al. [19] used ethylenediaminetetraacetic acid (EDTA) to chelate ZA from the bone matrix, rescuing osteoclast function, while Oizumi et al. [20] displaced potent BPs from the bone matrix with low-potency BPs in MRONJ patients, which promoted the recovery of soft tissues. A recent in vitro study demonstrated that calcium phosphate-based materials could bind ZA from solution, reducing cytotoxicity in oral fibroblasts [21]. Hydroxyapatite (HA) is a calcium phosphate-based bone filler material, known to support bone regeneration, which is currently approved for use in tooth socket repair [22]. In this study, we hypothesised that HA could bind free BPs, reducing local BP concentrations and preventing soft tissue toxicity associated with MRONJ development. The focus of our work was to measure BP toxicity to the oral mucosa in vitro, in both 2D and 3D, and to determine whether HA could inhibit the soft tissue toxicity caused by BPs.

## 2. Materials and Methods 

### 2.1. Microscopy 

PermaBone^®^ 100% porous HA was kindly provided from Ceramisys Limited (Sheffield, UK) in the forms of 1–4 mm porous granules (over 80% porosity) and pre-granulated material. The granules were examined using a stereomicroscope (Zeiss SteREO.V8 with a Zeiss SteREO CL1500 ECO light source, Oberkochen, Germany) and photographed using a 1.4 megapixel camera (Zeiss ICc 1, Oberkochen, Germany).

Pre-granulated HA was examined using scanning electron microscopy (SEM). The HA was placed on a carbon tab, gold coated by sputtering and imaged using a scanning electron microscope (Philips XL-20, Amsterdam, The Netherlands), operated at 13 kV.

### 2.2. Cell Culture

Human oral fibroblasts were isolated from human oral tissue through overnight incubation in 0.5 mg/mL collagenase A (Roche, Basel, Switzerland), and cultured in Dulbecco’s modified Eagle’s medium (DMEM) containing 0.01 mg/mL Penicillin/Streptomycin (P/S) and 0.01 mg/mL L-Glutamine (LG) (all Sigma-Aldrich, Dorset, UK) and 10% foetal calf serum (FCS) (Biosera, East Sussex, UK). All subjects gave their informed consent for inclusion before they participated in the study. The study was conducted in accordance with the Declaration of Helsinki, and the protocol was approved by the University of Sheffield Ethics Committee on 1st June 2015 (Reference number 003463). 

Human oral keratinocytes, immortalised through transduction (OKF6/TERT 2) [23], were cultured in keratinocyte serum-free medium with 0.05 mg/mL bovine pituitary extract and 0.005 µg/mL epidermal growth factor (EGF) (all Gibco, Paisley, UK) and 0.01 mg/mL P/S (Sigma-Aldrich). These cells were originally isolated by Lindberg and Rheinwald [24] and provided by Dr. James G Rheinwald. The 3D cell culture took place in Green’s medium—comprised of DMEM and Ham’s F12 nutrient mixture (Sigma-Aldrich) (3:1 ratio) with 0.01 mg/mL P/S, 0.01 mg/mL LG, 5 µg/mL insulin, 5 µg/mL apo transferrin, 4 µg/mL hydrocortisone, 0.625 µg/mL amphotericin B, 0.025 µg/mL adenine, 8.47 ng/mL cholera toxin, 5 ng/mL EGF and 1.36 ng/mL 3,3,5 tri-iodothyronine (all Sigma-Aldrich) and 10% FCS (BioSera).

### 2.3. De-Cellularised Dermis Preparation

Split-thickness skin was incubated at 37 °C in 1 M NaCl solution (Sigma-Aldrich) for 24 h and the epithelium was removed to produce de-cellularised dermis (DCD). All subjects gave their informed consent for inclusion before they participated in the study. The study was conducted in accordance with the Declaration of Helsinki, and the protocol was approved by the NHS Research Ethics Committee in 2015 (Amended 14 November 2019) (Reference number 15/YH/0177).

### 2.4. Bisphosphonates

Pamidronate disodium salt hydrate (PA) (Sigma-Aldrich) and zoledronic acid monohydrate (ZA) (Sigma-Aldrich) were dissolved in distilled water, aliquoted and stored at −20 °C. Clinically relevant concentrations of BPs were selected based on Scheper et al. [25]. 

### 2.5. Two-Dimensional Cell Viability Assays

The 3-(4,5-dimethylthiazol-2-yl)-2,5-diphenyltetrazolium bromide (MTT) (Sigma-Aldrich) assay was used to measure cell viability as per the manufacturer’s instructions. Fibroblasts (1.0 × 10^4^ cells per cm^2^) and keratinocytes (1.67 × 10^4^ cells per cm^2^) were seeded and left overnight to adhere. Cells were then treated with PA- or ZA-containing medium for up to 72 h. Values were normalised to 0 µM at 24 h.

Half maximal inhibitory concentrations (IC_50_) were calculated based on the MTT data. Values were first normalised to the control at 72 h, where concentrations were transformed to log_10_(concentration) with 0 µM set as 1 × 10^−10^ µM, and an IC_50_ was generated.

### 2.6. Three-Dimensional Cell Culture

Models of the oral mucosa were produced as described previously [26]. Briefly, DCD was cut into 400 mm^2^ pieces. Fibroblasts (2.5 × 10^5^) and keratinocytes (1 × 10^6^) were seeded on the DCD in stainless steel rings. At 0 h the medium was a DMEM and KSFM mixture (1:1), at 24 h half of this was replaced by Green’s medium, at 48 h the medium was entirely replaced by a KSFM and Green’s medium mixture (1:1). After seeding for 72 h, the rings were removed, models were quartered and placed onto a stainless steel grid at air–liquid interface (ALI) in Green’s medium. 

A resazurin reduction assay was performed to measure the metabolic activity of the oral mucosa after 7 days at ALI. Models were then cultured in a BP-containing medium and the resazurin assay was repeated at days 10 and 14. Models were incubated with resazurin sodium salt (Sigma Aldrich) (25.1 µg/mL) for 4 h before absorbance readings were taken (absorbance = 562 nm/reference = 630 nm). Samples were then washed with PBS and placed back at ALI. Values were normalised to those of each individual model at day 7. At day, 14 samples were fixed in 3.7% formaldehyde (Sigma-Aldrich) and analysed using standard histology. Photoshop CS2 (Adobe, San Jose, CA, USA) was used to digitally remove the background from images.

### 2.7. Hydroxyapatite Binding Assays

A 0.65 g amount of PermaBone^®^ granules (Ceramisys Limited, Sheffield, UK) was sterilised by autoclave and incubated with a BP-containing medium for 72 h. Cells were treated with a BP-containing medium or a medium incubated with HA and BPs for 72 h and an MTT assay was performed.

Oral mucosa models were seeded as above, halved with a sterile scalpel and placed at ALI for 7 days. Models were treated with either 0 µM control medium or 30 µM ZA from day 7. A 0.65 g amount of HA granules was placed under the stainless steel grid and a resazurin assay was performed at days 10 and 14. Samples were then fixed, processed for histology and stained as above.

### 2.8. Statistics

Prism 7 (GraphPad, San Diego, CA, USA) was used to perform all statistical analyses. Statistical significance was defined as p ≤ 0.05. Standard deviation (SD) was used for all error bars. For the 2D and 3D BP viability tests, repeated measures two-way analysis of variance (ANOVA) tests and Dunnett’s multiple comparison tests were carried out to investigate differences between BPs and control medium (0 µM) at each time point. One value, identified as a significant outlier (p ≤ 0.001) using Grubb’s test, was removed from the MTT data. In the HA experiments, post-hoc analysis was performed using Tukey’s test. Significance was calculated between the control group and each HA group, comparing the individual BP concentration of the controls with their corresponding concentration in the HA group (e.g., control 100 µM PA vs. HA granules 100 µM PA).

## 3. Results

### 3.1. Microscopy of Hydroxyapatite Granules Confirmed Highly Porous Structure

**Figure 1:**
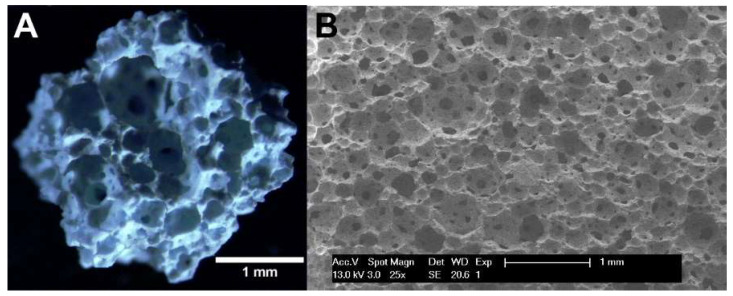
(**A**) A stereomicroscopy image of a PermaBone^®^ hydroxyapatite granule. (**B**) A scanning electron microscopy image of pre-granulated PermaBone^®^ hydroxyapatite.

The HA granules were examined under a stereomicroscope, and pre-granulated HA examined with SEM to examine their structure. The HA had an open porosity, with macropores of a range of diameters visible throughout the material (Figure 1).

### 3.2. Clinically Relevant Pamidronic Acid and Zoledronic Acid Concentrations are Toxic to Oral Mucosa Cells in 2D

**Figure 2:**
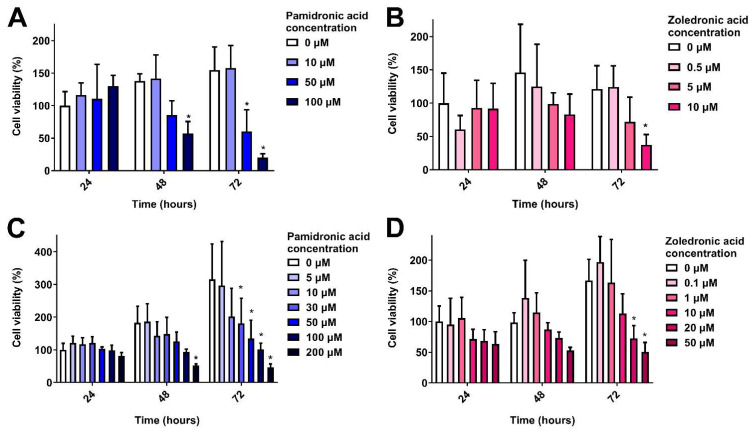
Cell metabolic activity over 72 h in the presence of bisphosphonates measured by MTT assay. (**A**) Human oral fibroblasts with pamidronic acid, (**B**) human oral fibroblasts with zoledronic acid, (**C**) immortalised human oral keratinocytes with pamidronic acid and (**D**) immortalised human oral keratinocytes with zoledronic acid. Values normalised to 0 µM at 24 h. One significant outlying value removed. N = 3, n = 3. Error bars = standard deviation (SD). Significance against 0 µM at each time point indicated by * *p* ≤ 0.05.

The toxicity of PA and ZA was first measured in 2D. Figure 2A shows the response of oral fibroblasts when cultured in different concentrations of PA over 72 h, normalised to the control (0 µM) value at 24 h. At 24 h, there were no significant differences in metabolic activity. At 48 h, 100 µM PA significantly reduced metabolic activity. Both 50 and 100 µM PA significantly lowered fibroblast metabolic activity after 72 h. The IC_50_ at 72 h was 43 µM.

When immortalised keratinocytes (OKF6) were cultured in PA, there were no significant differences at 24 h (Figure 2B). After 48 h, a dose response was seen, with 100 µM PA significantly reducing metabolic activity. PA concentrations of 30 µM and above significantly lowered keratinocyte metabolic activity after 72 h in a dose-dependent manner, and the IC_50_ was 35 µM.

ZA is a more potent anti-resorptive compared to PA [4] and was shown to be more toxic to oral mucosa cells. A dose-dependent response was seen with fibroblasts (Figure 2C), with 10 µM significantly reducing metabolic activity following 72 h culture, and an IC_50_ of 6 µM.

Figure 2D shows the response of immortalised keratinocytes to ZA. Again, no significance was found between doses at 24 or 48 h. At 72 h, concentrations of 20 and 50 µM significantly reduced cell metabolic activity in comparison to the control. The IC_50_ was 19 µM.

### 3.3. Pamidronic Acid and Zoledronic Acid Reduce Epithelial Thickness and Cause Toxicity to the Oral Mucosa in 3D

**Figure 3:**
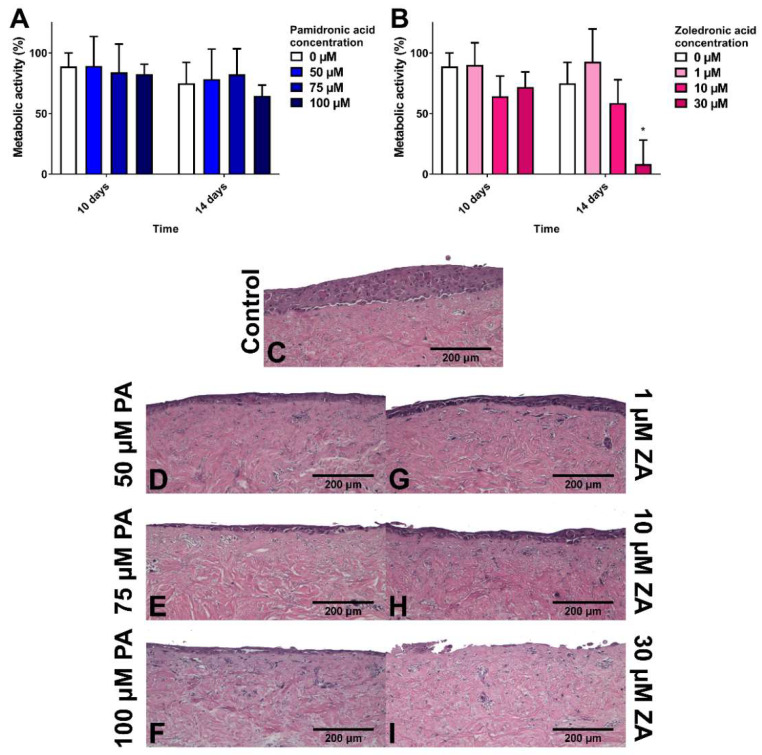
The metabolic activity of oral mucosa models when treated with (**A**) pamidronic acid and (**B**) zoledronic acid for 7 days after prior culture at ALI in control medium for 7 days, measured with a resazurin assay. A blank well reading was subtracted before values were normalised to day 7 value for each model, defining day 7 values as 100 % (not shown). N = 3, n = 3. Error bars = SD. Statistical significance against 0 µM at each time point indicated by * *p* ≤ 0.05. (**C**–**I**) H & E-stained sections of oral mucosa models seeded with human oral fibroblasts and immortalised human oral keratinocytes cultured at ALI for 7 days in control medium, then treated with (**C**) control medium; (**D**) 50 µM, (**E**) 75 µM and (**F**) 50 µM pamidronic acid; (**G**) 1 µM, (**H**) 10 µM and (**I**) 30 µM zoledronic acid, respectively, for 7 days. Representative images used.

To study how BPs affect healthy oral mucosa, models were cultured for 7 days to allow epithelial stratification before treating with BPs for 7 days. Figure 3A shows that PA had no significant effect on the metabolic activity of the models in comparison to the control. Values for all samples, including the control, decreased from day 7 to day 14. Figure 3C shows the epithelial stratification after 14 days in control medium. Figure 3D–F demonstrate that while PA caused a dose-dependent reduction in epithelial thickness, differentiated keratinocytes were still present in the superficial layer.

ZA concentrations of 1 and 10 µM had no metabolic effect, while a 30 µM concentration significantly lowered metabolic activity to approximately 10% after 7 days of treatment (Figure 3B). Figure 3G,H demonstrate that 1 and 10 µM ZA reduced epithelial thickness in a dose-dependent manner. No epithelium was present after 30 µM ZA treatment (Figure 3I).

### 3.4. Hydroxyapatite Granules Prevent Pamidronic Acid and Zoledronic Acid Toxicity in 2D

**Figure 4:**
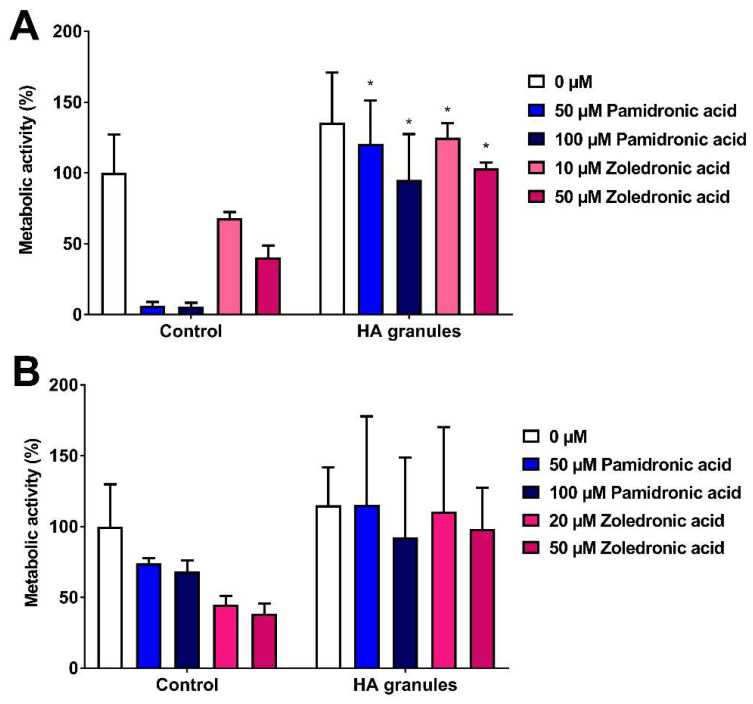
(**A**) Human oral fibroblast and (**B**) immortalised human oral keratinocyte metabolic activity over 72 h in the presence of pamidronic acid and zoledronic acid, measured by MTT assay. Bisphosphonates had previously been incubated for 72 h with hydroxyapatite (HA) granules; bisphosphonates incubated with no hydroxyapatite used as a control. Values normalised to control 0 µM. N = 3, n = 3. Error bars = SD. Significance against control indicated by * *p* ≤ 0.05.

As the toxicity limits were now defined, the ability of commercially available HA granules to reduce BP toxicity was examined. Figure 4A shows the metabolic activity of human oral fibroblasts treated with media containing PA or ZA that had been previously incubated with or without HA granules. PA and ZA were toxic to fibroblasts but pre-incubation of a BP-containing medium with HA granules inhibited BP toxicity to fibroblasts, as shown by significantly higher metabolic activities in comparison to the controls. There were no significant differences between untreated (0 µM) control cells and the untreated cells cultured with HA, indicating that the HA granules themselves were not affecting metabolic activity. 

Figure 4B shows the metabolic activity of immortalised keratinocytes following the same treatment as above. In the control group (no HA), dose-dependent toxicity was seen after 72 h, with both drugs reducing metabolic activity. HA granules inhibited this reduction in metabolic activity. However, variation was high, and no significant differences were seen between any HA groups and their corresponding control groups.

### 3.5. Hydroxyapatite Prevents Zoledronic Acid Toxicity in 3D

To assess HA effects on BP toxicity in a closer representation of the in vivo scenario, three-dimensional oral mucosa models were cultured. Oral mucosa models were cultured for 7 days at ALI before being treated with 30 µM ZA in the presence of HA granules; models with no HA present were used as controls.

**Figure 5:**
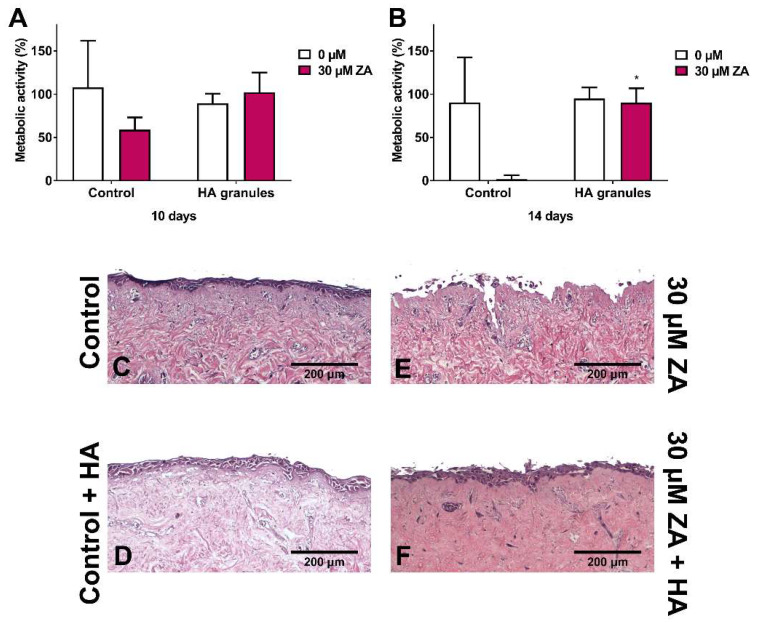
The metabolic activity of oral mucosa models when treated with control medium or 30 µM zoledronic acid in the presence of hydroxyapatite granules at (**A**) 10 days and (**B**) 14 days. Models with no HA cultured as controls, measured with a resazurin assay. Blank well reading subtracted from values before normalising to day 7 values for each model, defining day 7 values as 100 % (not shown). N = 3, n = 2. Error bars = SD. Statistical significance against control indicated by * *p* ≤ 0.05. (**C**–**F**) H & E-stained sections of oral mucosa models seeded with human oral fibroblasts and primary human oral keratinocytes cultured at ALI for 7 days in control medium, then treated with (**C**) control medium, (**E**) 30 µM zoledronic acid, (**D**) control medium and HA granules and (**F**) 30 µM zoledronic acid and HA granules, respectively, for 7 days. Representative images used.

Figure 5A shows the metabolic activity of oral mucosa models treated with ZA, with and without HA granules, after 10 days in culture. Metabolic activity values were normalised to day 7 values (before BP and HA treatment). As expected, treatment of the models with 30 µM ZA but without HA reduced the metabolic activity by approximately 50%. However, the models cultured with both ZA and HA had a metabolic activity close to 100%. 

At day 14 (Figure 5B), the metabolic activity of the control models remained at approximately 90% and treatment with 30 µM ZA reduced metabolic activity to <5%. Culture with HA granules and 30 µM ZA resulted in a significantly higher metabolic activity than those treated with the drug alone (approximately 90%).

Further analysis of these models showed that a multi-layered epithelium was present at 14 days when cultured in control media (Figure 5C) or media containing HA granules (Figure 5D). Models treated with 30 µM ZA had no epithelium remaining (Figure 5E). However, when HA granules were present, a stratified epithelium was present across the sample (Figure 5F), with some damage to the superficial layer.

## 4. Discussion

We have demonstrated that clinically relevant concentrations of PA and ZA reduce the metabolic activity of cells from the human oral mucosa in a dose-dependent manner. Our data showed that both fibroblasts and keratinocytes were negatively affected by BPs. This supports the hypothesis that BPs impair soft tissue wound healing and reduce cell viability, which in turn contributes to the development of MRONJ. 

PA and ZA have previously been demonstrated to be toxic to human oral fibroblasts and keratinocytes, with ZA showing more cytotoxicity [10,11,12,13,16,27,28,29,30,31,32,33,34]. However, these studies used cells from a variety of sources, examined different concentration ranges and treatment lengths, and used different assays to measure viability and therefore defining a toxic concentration for these drugs is difficult. Here, we have performed a comprehensive study of the toxicity of two BPs most commonly associated with MRONJ to allow for a more direct comparison between two drugs and two different cell types. The concentration of BPs the oral mucosa is exposed to in the clinical situation is difficult to measure. However, it has been estimated that the concentration of PA in bone is 2000 µM [35] and the salivary ZA concentration of patients with osteonecrosis is between 0.4 and 5 µM [25]. We have tested values within this range and demonstrated that concentrations toward the lower end can cause significant soft tissue cytotoxicity, and defined IC_50_ values that will be important in defining limits for the reduction in BPs in the treatment of MRONJ. 

In our in vitro system, we cultured oral mucosa models for 7 days to generate an established, multi-layer epithelium representative of the oral mucosa prior to MRONJ. PA and ZA treatments caused the established epithelia to thin in a dose-dependent manner that was in line with previous studies which showed that 10 µM PA and 4 to 10 µM ZA reduced epithelial thickness in oral mucosa models [15,16,18,32]. In addition to this morphological observation, we were able to measure the reduction in metabolic activity to quantify the negative effects that these drugs have on cell viability. As well as reducing epithelial thickness, we observed disruption to the integrity of the epithelium, which may be a result of changes in epithelial cell adhesion. A reduction in desmoglein-1 expression and disruption to desmosome morphology has previously been observed in the oral mucosa of patients receiving alendronate [36].

BPs have been found in the saliva of patients receiving BP therapy and, following dental interventions, it is expected that some BPs bound to the bone matrix will be released into the local oral environment, increasing the local concentration of BPs to which the oral mucosa will be exposed [37]. Here, we have shown that exposure to aqueous BPs leads to a thinner more fragile epithelium with reduced metabolic activity. These changes will reduce the oral mucosa’s ability to repair following damage and may explain how spontaneous cases of MRONJ occur [38], as the epithelium is more susceptible to damage from mechanical forces and infection. 

Hydroxyapatite is an osteoconductive material currently used for bone regeneration applications including socket repair. Given the high affinity of BPs to calcium, it was hypothesised that HA (a form of calcium phosphate) could bind BPs found in the oral cavity, reducing the bioavailability of these drugs and limiting their damage to surrounding soft tissues. We demonstrated that incubation with HA granules reduced fibroblast toxicity from ZA and PA, in line with recent work by Paulo et al. [21]. PermaBone^®^ granules were used, as they are clinically available, highly porous and comprised of pure HA and, therefore, due to the well reported BP binding relationship [5,6], it was thought they would bind BPs. The porosity of PermaBone^®^ HA that results in a high surface area can be seen in Figure 1—this maximised the amount of BP that can bind to the HA. Incubation with the same weight of HA in a pressed, sintered disc rather than in porous granules did not show the same effect (data not shown), demonstrating that surface area is important and supporting the hypothesis that BPs bind to the surface of the HA, rendering them incapable of exerting their damaging effects. 

When immortalised keratinocytes were cultured in medium that had been incubated with HA granules, there was large variation in the data, particularly in those also treated with PA and ZA. Extracellular calcium concentration is known to affect differentiation, morphology and proliferation in skin keratinocytes [39]. In addition, calcium and ZA have been shown to have a synergistic effect on skin keratinocyte viability, as higher cellular calcium caused increased BP potency [27]. Therefore, the variations observed in the data here are thought to be as a result of changes in calcium levels following incubation with HA granules. Despite these variations, the metabolic activity of keratinocytes was higher in all the samples incubated with HA granules compared to controls, so we proceeded to test this in the 3D oral mucosa models.

When oral mucosa models were cultured in the presence of ZA, HA granules provided a protective effect. The metabolic rate of cells in the oral mucosa model treated with HA and ZA were comparable to the models without ZA treatment, while histology showed that the epithelial integrity was intact. There was some damage to the superficial layers of the epithelium. However, the addition of HA granules resulted in a clear improvement compared to the epithelium treated with ZA alone, which was entirely lost. 

This study has demonstrated that HA granules can bind sufficient quantities of BPs to reduce soft tissue damage, which indicates its potential as a preventative therapy for MRONJ. Further research is now required to determine the most clinically feasible formulation of HA for this application and to evaluate how BP adsorption affects HA granule resorption and bone regeneration. 

## 5. Conclusions

We have demonstrated that hydroxyapatite can be used to inhibit BP toxicity to the oral mucosa in vitro. The application of porous HA granules to reduce BP concentrations following oral surgery presents a potential strategy for the prevention of MRONJ in high-risk patients receiving nitrogen containing BPs such as ZA and PA.

## References

[1] Dimopoulos M.A., Kastritis E., Anagnostopoulos A., Melakopoulos I., Gika D., Moulopoulos L.A., Bamia C., Terpos E., Tsionos K., Bamias A. (2006). Osteonecrosis of the jaw in patients with multiple myeloma treated with bisphosphonates: Evidence of increased risk after treatment with zoledronic acid. Haematologica.

[2] Ruggiero S.L., Dodson T.B., Fantasia J., Goodday R., Aghaloo T., Mehrotra B., O’Ryan F. (2014). American Association of Oral and Maxillofacial Surgeons position paper on medication-related osteonecrosis of the jaw—2014 update. J. Oral Maxillofac. Surg..

[3] Khosla S., Burr D., Cauley J., Dempster D.W., Ebeling P.R., Felsenberg D., Gagel R.F., Gilsanz V., Guise T., Koka S. (2007). Bisphosphonate-associated osteonecrosis of the jaw: Report of a task force of the American Society for Bone and Mineral Research. J. Bone Miner. Res..

[4] Ebetino F.H., Hogan A.-M.L., Sun S., Tsoumpra M.K., Duan X., Triffitt J.T., Kwaasi A.A., Dunford J.E., Barnett B.L., Oppermann U. (2011). The relationship between the chemistry and biological activity of the bisphosphonates. Bone.

[5] Russell R.G.G. (2006). Bisphosphonates: From bench to bedside. Ann. N. Y. Acad. Sci..

[6] Paulo S., Abrantes A.M., Laranjo M., Carvalho L., Serra A., Botelho M.F., Ferreira M.M. (2014). Bisphosphonate-related osteonecrosis of the jaw: Specificities. Oncol. Rev..

[7] Barba-Recreo P., de Vera J.L.d.P., Georgiev-Hristov T., Bravo-Burguillos E.R., Abarrategi A., Burgueño M., García-Arranz M. (2015). Adipose-derived stem cells and platelet-rich plasma for preventive treatment of bisphosphonate-related osteonecrosis of the jaw in a murine model. J. Cranio-Maxillofac. Surg..

[8] Galis B., Zajko J., Hirjak D., Vanko L., Kupcova I., Jurkemik J., Gengelova P., Mikuskova K., Halmova K., Riznic M. (2017). Is the Prevalence of the Medication-Related Osteonecrosis of the Jaws Underestimated, Evaluation in Oncological and Non-Oncological Disease. Bratisl. Med. J..

[9] Reid I.R., Bolland M.J., Grey A.B. (2007). Is bisphosphonate-associated osteonecrosis of the jaw caused by soft tissue toxicity?. Bone.

[10] Cozin M., Pinker B.M., Solemani K., Zuniga J.M., Dadaian S.C., Cremers S., Landesberg R., Raghavan S. (2011). Novel therapy to reverse the cellular effects of bisphosphonates on primary human oral fibroblasts. J. Oral Maxillofac. Surg..

[11] Hagelauer N., Ziebart T., Pabst A.M., Walter C. (2015). Bisphosphonates inhibit cell functions of HUVECs, fibroblasts and osteogenic cells via inhibition of protein geranylgeranylation. Clin. Oral Investig..

[12] Pabst A.M., Ziebart T., Koch F.P., Taylor K.Y., Al-Nawas B., Walter C. (2012). The influence of bisphosphonates on viability, migration, and apoptosis of human oral keratinocytes-in vitro study. Clin. Oral Investig..

[13] Scheper M.A., Badros A., Chaisuparat R., Cullen K.J., Meiller T.F. (2009). Effect of zoledronic acid on oral fibroblasts and epithelial cells: A potential mechanism of bisphosphonate-associated osteonecrosis. Br. J. Haematol..

[14] Renò F., Migliario M., Rizzi M., Invernizzi M., Cisari C., Cannas M. (2012). Low concentration amino-bisphosphonates stimulate human keratinocyte proliferation and in vitro wound healing. Int. Wound J..

[15] Bae S., Sun S., Aghaloo T., Oh J.-E., McKenna C.E., Kang M.K., Shin K.-H., Tetradis S., Park N.-H., Kim R.H. (2014). Development of oral osteomucosal tissue constructs in vitro and localization of fluorescently-labeled bisphosphonates to hard and soft tissue. Int. J. Mol. Med..

[16] Kim R.H., Lee R.S., Williams D., Bae S., Woo J., Lieberman M., Oh J.-E., Dong Q., Shin K.-H., Kang M.K. (2011). Bisphosphonates Induce Senescence in Normal Human Oral Keratinocytes. J. Dent. Res..

[17] McLeod N.M.H., Moutasim K.A., Brennan P.A., Thomas G., Jenei V. (2014). In vitro effect of bisphosphonates on oral keratinocytes and fibroblasts. J. Oral Maxillofac. Surg..

[18] Saito T., Izumi K., Shiomi A., Uenoyama A., Ohnuki H., Kato H., Terada M., Nozawa-Inoue K., Kawano Y., Takagi R. (2014). Zoledronic acid impairs re-epithelialization through down-regulation of integrin αvβ6 and transforming growth factor beta signalling in a three-dimensional in vitro wound healing model. Int. J. Oral Maxillofac. Surg..

[19] Elsayed R., Abraham P., Awad M.E., Kurago Z., Baladhandayutham B., Whitford G.M., Pashley D.H., McKenna C.E., Elsalanty M.E. (2018). Removal of matrix-bound zoledronate prevents post-extraction osteonecrosis of the jaw by rescuing osteoclast function. Bone.

[20] Oizumi T., Yamaguchi K., Sato K., Takahashi M., Yoshimura G., Otsuru H., Tsuchiya M., Hagiwara Y., Itoi E., Sugawara S. (2016). A Strategy against the Osteonecrosis of the Jaw Associated with Nitrogen-Containing Bisphosphonates (N-BPs): Attempts to Replace N-BPs with the Non-N-BP Etidronate. Biol. Pharm. Bull..

[21] Paulo S., Laranjo M., Abrantes A.M., Casalta-Lopes J., Santos K., Gonçalves A.C., Paula A.B., Marto C.M., Sarmento-Ribeiro A.B., Carrilho E. (2019). Synthetic Calcium Phosphate Ceramics as a Potential Treatment for Bisphosphonate-Related Osteonecrosis of the Jaw. Materials.

[22] Kattimani V.S., Kondaka S., Lingamaneni K.P. (2016). Hydroxyapatite—Past, Present, and Future in Bone Regeneration. Bone Tissue Regen. Insights.

[23] Dickson M.A., Hahn W.C., Ino Y., Ronfard V., Wu J.Y., Weinberg R.A., Louis D.N., Li F.P., Rheinwald J.G. (2000). Human keratinocytes that express hTERT and also bypass a p16(INK4a)-enforced mechanism that limits life span become immortal yet retain normal growth and differentiation characteristics. Mol. Cell. Biol..

[24] Lindberg K., Rheinwald J.G. (1990). Three distinct keratinocyte subtypes identified in human oral epithelium by their patterns of keratin expression in culture and in xenografts. Differentiation.

[25] Scheper M.A., Badros A., Salama A.R., Warburton G., Cullen K.J., Weikel D.S., Meiller T.F. (2009). A novel bioassay model to determine clinically significant bisphosphonate levels. Supportive Care Cancer.

[26] Grayson A.K., Hearnden V., Bolt R., Jebreel A., Colley H.E., Murdoch C. (2018). Use of a Rho kinase inhibitor to increase human tonsil keratinocyte longevity for three-dimensional, tissue engineered tonsil epithelium equivalents. J. Tissue Eng. Regen. Med..

[27] Arai N., Inoue S., Tomihara K., Tsuno H., Noguchi M. (2013). In vitro synergistic effects of zoledronic acid and calcium on viability of human epithelial cells. Oral Dis..

[28] Açil Y., Arndt M.L., Gülses A., Wieker H., Naujokat H., Ayna M., Wiltfang J. (2018). Cytotoxic and inflammatory effects of alendronate and zolendronate on human osteoblasts, gingival fibroblasts and osteosarcoma cells. J. Cranio-Maxillofac. Surg..

[29] Agis H., Blei J., Watzek G., Gruber R. (2010). Is Zoledronate Toxic to Human Periodontal Fibroblasts?. J. Dent. Res..

[30] Draenert G.F., Huetzen D.O., Kämmerer P.W., Palarie V., Nacu V., Wagner W. (2012). Dexrazoxane shows cytoprotective effects in zoledronic acid-treated human cells in vitro and in the rabbit tibia model in vivo. J. Cranio-Maxillofac. Surg..

[31] Jung J., Park J.S., Righesso L., Pabst A.M., Al-Nawas B., Kwon Y.D., Walter C. (2018). Effects of an oral bisphosphonate and three intravenous bisphosphonates on several cell types in vitro. Clin. Oral Investig..

[32] Ohnuki H., Izumi K., Terada M., Saito T., Kato H., Suzuki A., Kawano Y., Nozawa-Inoue K., Takagi R., Maeda T. (2012). Zoledronic acid induces S-phase arrest via a DNA damage response in normal human oral keratinocytes. Arch. Oral Biol..

[33] Ravosa M.J., Ning J., Liu Y., Stack M.S. (2011). Bisphosphonate effects on the behaviour of oral epithelial cells and oral fibroblasts. Arch. Oral Biol..

[34] Soydan S.S., Araz K., Senel F.V., Yurtcu E., Helvacioglu F., Dagdeviren A., Tekindal M.A., Sahin F. (2015). Effects of alendronate and pamidronate on apoptosis and cell proliferation in cultured primary human gingival fibroblasts. Hum. Exp. Toxicol..

[35] Marolt D., Cozin M., Vunjak-Novakovic G., Cremers S., Landesberg R. (2012). Effects of pamidronate on human alveolar osteoblasts in vitro. J. Oral Maxillofac. Surg..

[36] Donetti E., Gualerzi A., Sardella A., Lodi G., Carrassi A., Sforza C. (2014). Alendronate impairs epithelial adhesion, differentiation and proliferation in human oral mucosa. Oral Dis..

[37] Ikebe T. (2013). Pathophysiology of BRONJ: Drug-related osteoclastic disease of the jaw. Oral Sci. Int..

[38] Khominsky A., Lim M. (2018). “Spontaneous” medication-related osteonecrosis of the jaw; two case reports and a systematic review. Aust. Dent. J..

[39] Micallef L., Belaubre F., Pinon A., Jayat-Vignoles C., Delage C., Charveron M., Simon A. (2009). Effects of extracellular calcium on the growth-differentiation switch in immortalized keratinocyte HaCaT cells compared with normal human keratinocytes. Exp. Dermatol..

